# Associations between systemic bone mineral density and early knee cartilage changes in middle-aged adults without clinical knee disease: a prospective cohort study

**DOI:** 10.1186/s13075-017-1314-0

**Published:** 2017-05-18

**Authors:** Andrew J. Teichtahl, Yuanyuan Wang, Anita E. Wluka, Boyd J. Strauss, Joseph Proietto, John B. Dixon, Graeme Jones, Flavia M. Cicuttini

**Affiliations:** 1 Department of Epidemiology and Preventive Medicine, School of Public Health and Preventive Medicine, Monash University, Alfred Hospital, Melbourne, VIC 3004 Australia; 2 Baker IDI Heart and Diabetes Institute, Commercial Road, Melbourne, VIC 3004 Australia; 3 Department of Medicine, Monash University, Melbourne, VIC 3168 Australia; 4 Department of Medicine, University of Melbourne, Austin Health, Melbourne, VIC 3050 Australia; 5 Menzies Research Institute, Tasmania, Private bag 23, Hobart, TAS 7000 Australia

**Keywords:** Bone mineral density, Cartilage volume, Cartilage defects, Osteoarthritis, Knee

## Abstract

**Background:**

Osteoarthritis has a high prevalence in people with high bone mineral density (BMD). Nevertheless, whether high systemic BMD predates early structural features of knee osteoarthritis is unclear. This study examined the association between systemic BMD and knee cartilage defect progression and cartilage volume loss in middle-aged people without clinical knee disease.

**Methods:**

Adults (*n* = 153) aged 25–60 years had total body, lumbar spine, and total hip BMD assessed by dual-energy X-ray absorptiometry at baseline (2005–2008), and tibial cartilage volume and tibiofemoral cartilage defects assessed by magnetic resonance imaging at baseline and follow up (2008–2010).

**Results:**

Higher spine BMD was associated with increased risk for progression of medial (OR = 1.45, 95% CI 1.10, 1.91) and lateral (OR = 1.30, 95% CI 1.00, 1.67) tibiofemoral cartilage defects. Total hip BMD was also positively associated with the progression of medial (OR = 1.63, 95% CI 1.10, 2.41) and lateral (OR = 1.53, 95% CI 1.08, 2.18) tibiofemoral cartilage defects. Greater total body, spine, and total hip BMD were associated with increased rate of lateral tibial cartilage volume loss (for every 1 g/10 cm^2^ increase in total body BMD: B = 0.44%, 95% CI 0.17%, 0.71%; spine BMD: 0.17%, 95% CI 0.04%, 0.30%; total hip BMD: 0.29%, 95% CI 0.13%, 0.45%), with no significant associations for medial tibial cartilage volume loss.

**Conclusion:**

In middle-aged people without clinical knee disease, higher systemic BMD was associated with increased early knee cartilage damage. Further work is needed to clarify the effect of systemic BMD at different stages of the pathway from health through to disease in knee osteoarthritis, as new therapies targeting bone are developed for the management of knee osteoarthritis.

## Background

Bone is considered an integral structure in the pathogenesis of osteoarthritis (OA) and the role of local and systemic bone mineral density (BMD) is gaining increasing interest. When the knee joint is examined for OA outcomes, local BMD refers to subchondral or periarticular BMD of the tibia and systemic BMD refers to BMD of the hip, lumbar spine, and total body [1, 2]. It has been speculated that OA is more prevalent in people with higher systemic BMD [3, 4] and that there is an inverse relationship between osteoporosis and OA [3, 5, 6]. Higher systemic BMD may not reflect better-quality bone, as higher lumbar spine BMD is associated with lumbar spondylosis [7]. Such associations are thought to be due to either higher BMD within sclerotic areas, or generalized increase in subchondral bone, both of which are features that characterize knee OA [8–10]. With the advent of medications that modify bone turnover, a better understanding of the relationship between systemic BMD and early structural changes in knee OA may have important implications for disease onset and or progression. Indeed, a large radiographic study of 1754 participants demonstrated that high systemic BMD increases the risk of incident knee OA, as measured by the onset of joint space narrowing [2]. However, radiographic joint space narrowing provides only a surrogate measure of cartilage, with magnetic resonance imaging (MRI) evidence that approximately 11–13% of cartilage volume has been lost prior to radiographic evidence of any diminution of the joint space [11]. Cartilage volume loss and cartilage defects are both clinically significant as they are associated with the important patient outcomes of pain [12, 13] and risk of knee replacement [14, 15].

There has been increasing interest in the relationship between systemic BMD and cartilage properties since 2010, in particular in MRI studies (summarized in Table 1). Two cross-sectional studies demonstrated that systemic BMD is positively associated with knee cartilage volume in predominantly asymptomatic or healthy middle-aged populations [16, 17], with one study also showing a positive association between systemic BMD and cartilage defects [16]. As greater cartilage volume or thickness may indicate cartilage swelling in the early stages of degeneration rather than more healthy cartilage in the setting of early OA [18], an interpretation of results from the cross-sectional studies [16, 17] is that higher systemic BMD is associated with the early cartilage changes of knee OA. In the only longitudinal MRI study of people with and without radiographic knee OA, there was no significant association between baseline systemic BMD and change in cartilage volume or thickness in a modest number of people without knee OA (*n* = 69) [1]. There has been no longitudinal examination of whether systemic BMD may be associated with earlier changes in articular cartilage, such as the progression of cartilage defects, in populations without clinical knee disease.

**Table 1:** Studies since 2010 examining the association between systemic bone mineral density (BMD) and tibiofemoral cartilage

Author year	Study population	BMD site and value (mean (SD), g/cm^2^)	Main results
Cross-sectional study			
Brennan 2011 [16]	*N* = 160 (100% female) Asymptomatic participants Age 29–50 years, mean (SD) 41.4 (5.3) years BMI 27.6 (6.4) kg/m^2^	Spine 1.263 (0.150) Total body 1.178 (0.078) Femoral neck 1.008 (0.137) Ward’s triangle 0.915 (0.152) Trochanter 0.840 (0.112) Ultra-distal forearm 0.331 (0.043) Mid forearm 0.712 (0.052)	Spine, total body, and femoral neck BMD were positively associated with medial and lateral cartilage volume. Spine BMD was positively associated with the presence of medial compartment cartilage defects, and forearm BMD was positively associated with the prevalence of lateral compartment cartilage defects
Berry 2011 [17]	*N* = 153 (81% female) Predominantly healthy participants Age 25–60 years, mean (SD) men 46 (9), women 47 (10) years BMI, men 30 (8), women 33 (9) kg/m^2^	Total body, men 1.282 (0.10), women 1.228 (0.10)	Total body BMD was positively associated with medial and lateral tibial cartilage volume in men and women
Cohort study			
Nevitt 2010 [2]	*N* = 1754 (63.0% female) Participants with knee OA or at high risk of developing knee OA Age 50–79 years, mean (SD) 63.2 (7.8) years BMI 29.9 (5.4) kg/m^2^	Whole body, men 1.11 (0.11), women 0.94 (0.09) Femoral neck, men 0.84 (0.13), women 0.77 (0.12)	In knees without knee OA, higher femoral neck and whole body BMD were associated with increases in grade of joint space narrowing. In knees with existing knee OA, progression was not significantly related to BMD
Lee 2013 [26]	*N* = 127 (59% female) Symptomatic knee OA and Kellgren Lawrence grade ≥2 Age >45 years, mean (SD) 62.7 (8.6) years BMI 30.1 (5.4) kg/m^2^ 2 years follow up	Femoral neck 0.95 (0.14)	There were no significant associations between baseline BMD and cartilage volume or thickness Longitudinal BMD loss was associated with loss of femoral and tibial cartilage volume and thickness
Cao 2014 [1]	*N* = 158 (48% female) Randomly selected subjects; 69 without radiographic OA and 89 with radiographic OA Age mean (SD) 62.6 (7.2) years BMI 27.4 (4.1) kg/m^2^ 2.7 years follow up	Total body 1.08 (0.16) Total hip 0.98 (0.17) Spine 1.02 (0.20)	Cross-sectional analysis: total body, total hip, and spine BMD were positively associated with femoral and lateral tibial cartilage thickness in subjects with OA Longitudinal analysis: high total body BMD was associated with an increase in femoral cartilage thickness; high spine BMD was associated with increases in femoral and lateral tibial cartilage thickness in subjects with OA No significant associations were observed in subjects without OA

*BMI* body mass index, *OA* osteoarthritis

The aim of this prospective cohort study was to examine the associations between systemic BMD and early cartilage changes (change in cartilage volume and cartilage defects) in middle-aged adults with no clinical knee OA. We hypothesized that higher systemic BMD would be associated with deleterious cartilage outcomes in a pre-clinical cohort.

## Methods

### Study participants

A total of 153 participants, aged 25–60 years, were recruited to take part in a study of the relationship between obesity and musculoskeletal disease by advertising in the local press, at the hospitals in the waiting rooms of private weight loss/obesity clinics, and through community weight loss organisations in order to recruit participants across the spectrum from normal weight to obese (body mass index (BMI) ≥30 kg/m^2^) [17]. Participants were excluded if there was a history of any arthropathy diagnosed by a medical practitioner (including clinical OA as defined by the American College of Rheumatology criteria [19], inflammatory processes such as rheumatoid arthritis, or crystal arthropathies), prior surgical intervention to the knee, including arthroscopy, previous significant knee injury requiring non-weight-bearing therapy, knee pain precluding weight-bearing activity for >24 hours or requiring prescribed analgesia, malignancy, or contraindication to MRI. Baseline assessment was performed in 2005–2008, incorporating dual-energy X-ray absorptiometry (DXA), MRI, anthropometric measures, questionnaires about physical activity, and the Western Ontario and McMaster University Osteoarthritis Index (WOMAC), with MRI performed again at follow up in 2008–2010, an average of 2.3 (±0.4) years later. The study was approved by the Alfred Hospital Ethics Committee, Austin Health Human Research Ethics Committee, and Monash University Human Research Ethics Committee. All participants gave written informed consent.

### DXA

BMD (g/cm^2^) was measured using DXA (GE Lunar Prodigy, using operating system version 9) at the total body, lumbar spine (vertebrae L_2_ - L_4_), and total hip; BMD is calculated by dividing the bone mineral content by the area measured. The machine has a weight limit of ~130 kg. The coefficient of variation (CV) for BMD was 1.2–1.3% [17].

### MRI and knee structural assessments

MRI of the dominant knee (defined as the knee used when kicking a ball) was performed. Knees were imaged in the sagittal plane on a 1.5-T whole body magnetic resonance unit (Philips, Medical Systems, Eindhoven, the Netherlands) using a commercial transmit-receive extremity coil. The weight limit for the machine is 150 kg. The following sequence and parameters were used: T1-weighted fat saturation 3D gradient recall acquisition in the steady state (58 ms/12 ms/55°, repetition time/echo time/flip angle) with a 16-cm field of view, 60 partitions, 512 × 512 matrix and acquisition time 11 minutes 56 sec (one acquisition). Sagittal images were obtained at a partition thickness of 1.5 mm and an in-plane resolution of 0.31 × 0.31 mm. All MRI measurements were performed by trained observers who were blinded to participant characteristics and the sequence of images.

Tibial cartilage volumes were determined by manually drawing disarticulation contours around the cartilage boundary on T1-weighted sagittal images, using the Osiris software (Digital Imaging Unit, University Hospital of Geneva, Switzerland). Measurement was done by one trained observer with forty random cross checks blindly performed by an independent trained observer. The CV was 3.4% for the medial and 2.0% for the lateral tibial cartilage volume [20]. Annual percentage change in cartilage volume was calculated by (baseline cartilage volume − follow-up cartilage volume)/baseline cartilage volume/time between MRI scans *100. Thus a more positive value corresponds to greater cartilage volume loss.

Cartilage defects in the medial and lateral tibial and femoral cartilage were graded using a previously described classification system [21]: grade 0 = normal cartilage; grade 1 = focal blistering and intra-cartilaginous low-signal intensity area with an intact surface and bottom; grade 2 = irregularities on the surface or bottom and loss of thickness of less than 50%; grade 3 = deep ulceration with loss of thickness of more than 50%; grade 4 = full-thickness cartilage wear with exposure of subchondral bone. Intraobserver reliability (expressed as the intraclass correlation coefficient) was 0.90 for the medial tibiofemoral compartment and 0.89 for the lateral tibiofemoral compartment [21]. A prevalent cartilage defect was defined as a cartilage defect score ≥2 at either the medial or lateral tibiofemoral compartment. Progression of cartilage defects (versus stable defects and regression) was determined if there was an increase over time in the cartilage defect score in the medial or lateral tibiofemoral compartment.

The cross-sectional areas of the medial and lateral tibial plateau were measured from reformatted axial images using the Osiris software. The CV was 2.3% for measurement of the medial and 2.4% for measurement of the lateral tibial plateau area [22].

### Anthropometric data

Weight was measured at baseline to the nearest 0.1 kg (shoes, socks, and bulky clothing removed) using a single pair of electronic scales. Height was measured to the nearest 0.1 cm (shoes and socks removed) using a stadiometer. BMI (kg/m^2^) was calculated from these data.

### WOMAC

Pain, stiffness, and function were assessed at baseline by the WOMAC [23], which is widely used in community-based studies of adults. The pain, stiffness, and function subscales comprise 5, 2, and 17 questions, respectively. Each question is assessed on a 100-mm visual analogue scale and summed to give a total score out of 500 for pain, 200 for stiffness, and 1700 for function. An increase in the score corresponds with worsening of pain, stiffness, and functional difficulties. Participants were asked to rate their response for the knee that was examined by MRI.

### Strenuous physical activity

Strenuous physical activity was assessed by asking participants whether in the fortnight preceding MRI, they had participated in physical activity severe enough to raise their heart rate and cause diaphoresis for at least 20 minutes/day.

### Statistical analyses

With 139 participants completing the 2 year follow up, this study had 80% power to detect a correlation as low as 0.17 between BMD and cartilage volume loss (thus explaining up to 2.9% of the variance of cartilage volume loss), and to detect an odds ratio (OR) of 1.80 for progression of cartilage defects, with alpha = 0.05 and two-sided significance. Multiple linear regression analyses were used to determine the associations between systemic BMD and annual percentage change in tibial cartilage volume, adjusted for gender, baseline age, BMI, strenuous physical activity, and respective tibial bone area, in multivariable analyses. Binary logistic regression analyses were used to determine the associations between systemic BMD and the progression of tibiofemoral cartilage defects, adjusted for gender, baseline age, BMI, strenuous physical activity, respective baseline tibial cartilage volume and bone area, and time between MRI scans, in multivariable analyses. Menopausal status is another potential confounder, as systemic BMD in women differs according to the menopausal status. Our study did not collect data on menopausal status as part of the questionnaires. However, as menopause occurs on average around the age of 51 years [24], an age cutoff of 51 years can be used as a surrogate measure for menopausal status. In these analyses, BMD (g/cm^2^) was multiplied by 10 to convert to g/10 cm^2^ so that one unit change in BMD corresponded to approximately one standard deviation change in BMD. A *p* value <0.05 (two-tailed) was regarded as statistically significant. All analyses were performed using IBM SPSS Statistics for Windows (Version 23.0, IBM Corp, Armonk, NY, USA).

## Results

Participant characteristics are shown in Table 2. The cohort was predominantly female (81.7%), with a mean age of 46.7 ± 9.3 years and a mean BMI of 32.4 ± 8.9 kg/m^2^. More than half of the cohort (53.6%) was obese. No participant had osteoporosis (defined by a T- score ≤ −2.5), with 2.6–12.4% of the cohort having osteopenia (T-score between −1 and −2.5) based on their T-score at different body sites. There were 139 participants (91%) completing the follow up of the study and 14 participants were lost to follow up (9 participants withdrew, 4 were unable to be contacted and 1 relocated), with no significant differences in baseline characteristics between the two groups of participants.

**Table 2:** Participant characteristics

Characteristic	Value
Baseline	
Number of participants	153
Age, years	46.7 (9.3)
Female, *n* (%)	125 (81.7)
Body mass index, kg/m^2^	32.4 (8.9)
Range	16.9 to 54.9
Obesity, *n* (%)	82 (53.6)
WOMAC, median	
Pain (out of 500)	15
Stiffness (out of 200)	5
Function (out of 1700)	52
Strenuous physical activity, *n* (%)	119 (77.8)
Bone mineral density	
Total body	
Bone mineral density, g/cm^2^	1.24 (0.10)
T-score	1.18 (1.22)
T-score, range	−1.90 to 4.40
Osteopenia, *n* (%)/osteoporosis, *n* (%)	4 (2.6)/0 (0)
Spine	
Bone mineral density, g/cm^2^	1.26 (0.17)
T-score	0.46 (1.44)
T-score, range	−2.30 to 4.80
Osteopenia, *n* (%)/osteoporosis, *n* (%)	19 (12.4)/0 (0)
Total hip	
Bone mineral density, g/cm^2^	1.09 (0.16)
T-score	0.63 (1.29)
T-score, range	−2.40 to 4.50
Osteopenia, *n* (%)/osteoporosis, *n* (%)	13 (8.5)/0 (0)
Tibial cartilage volume, mm^3^	
Medial	996 (252)
Lateral	1299 (369)
Tibiofemoral cartilage defects, *n* (%)	
Medial	96 (62.7)
Lateral	135 (88.2)
Tibial bone area, mm^2^	
Medial	1888 (254)
Lateral	1455 (211)
Change	
Number of participants	139
Annual change in tibial cartilage volume, %	
Medial	1.5 (1.7)
Lateral	1.0 (1.5)
Progression of tibiofemoral cartilage defects, *n* (%)	
Medial	23 (16.5)
Lateral	26 (18.7)

Results displayed as mean (standard deviation) unless otherwise stated. *WOMAC* Western Ontario and McMaster University Osteoarthritis Index

The associations between systemic BMD at baseline and progression of tibiofemoral cartilage defects are presented in Table 3. In multivariable analyses, higher baseline spine BMD was associated with an increased risk of progression in medial and lateral tibiofemoral cartilage defects (OR 1.45, 95% CI 1.10, 1.91, *p* = 0.01, and OR 1.30, 95% CI 1.00, 1.67, *p* = 0.049, respectively). Total hip BMD was also positively associated with the risk of progression in medial and lateral tibiofemoral cartilage defects (OR 1.63, 95% CI 1.10, 2.41, *p* = 0.02, and OR 1.53, 95% CI 1.08, 2.18, *p* = 0.02, respectively). No significant associations were observed for total body BMD.

**Table 3:** Association between baseline systemic bone mineral density and progression of tibiofemoral cartilage defects

	Univariable analysis Odds ratio (95% CI)	*P*	Multivariable analysis^a^ Odds ratio (95% CI)	*P*
Total body BMD				
Medial	1.25 (0.80, 1.94)	0.33	1.71 (0.94, 3.08)	0.08
Lateral	1.19 (0.78, 1.81)	0.42	1.45 (0.84, 2.50)	0.18
Spine BMD				
Medial	1.38 (1.07, 1.77)	0.01	1.45 (1.10, 1.91)	0.01
Lateral	1.19 (0.94, 1.52)	0.15	1.30 (1.00, 1.67)	0.049
Total hip BMD				
Medial	1.33 (1.00, 1.75)	0.05	1.63 (1.10, 2.41)	0.02
Lateral	1.22 (0.93, 1.59)	0.15	1.53 (1.08, 2.18)	0.02

BMD (bone mineral density) was assessed in g/10 cm^2^ so that one unit change in BMD approximated one standard deviation change in BMD. ^a^Adjusted for gender, baseline age, body mass index, strenuous physical activity, respective baseline tibial cartilage volume and bone area, and time between scans

**Fig. 1:**
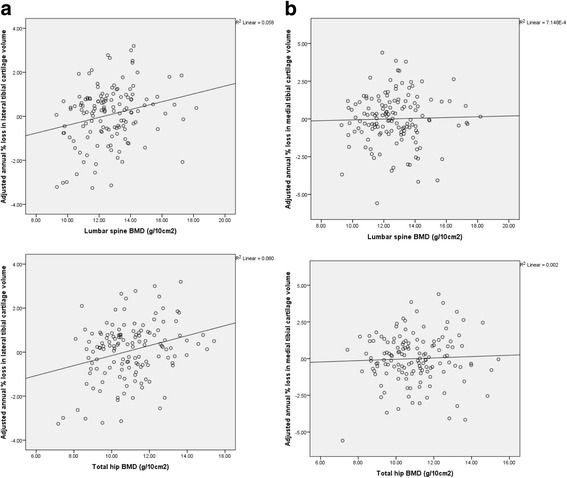
Association between baseline systemic bone mineral density (*BMD*; g/10 cm^2^) and annual percentage loss in tibial cartilage volume adjusted for age, gender, body mass index, strenuous physical activity, and respective tibial bone area. **a** Annual percentage loss in lateral tibial cartilage volume in relation to spine BMD (*r* = 0.24, *p* = 0.01) and total hip BMD (*r* = 0.28, *p* = 0.001); **b** Annual percentage loss in medial tibial cartilage volume in relation to spine BMD (*r* = 0.03, *p* = 0.75) and total hip BMD (*r* = 0.04, *p* = 0.53)

The associations between systemic BMD at baseline and tibial cartilage volume loss are presented in Table 4. In multivariable analyses, higher baseline total body BMD, spine BMD, and total hip BMD were all associated with an increased rate of lateral tibial cartilage volume loss (for every 1 g/10 cm^2^ increase in total body BMD: B = 0.44%, 95% CI 0.17%, 0.71%, *p* = 0.002; spine BMD: B = 0.17%, 95% CI 0.04%, 0.30%, *p* = 0.01; total hip BMD: B = 0.29%, 95% CI 0.13%, 0.45%, *p* = 0.001). No significant association was observed between systemic BMD measures and medial tibial cartilage volume loss. The relationship between spine and total hip BMD and tibial cartilage volume loss is also shown in Fig. 1.

**Table 4:** Association between baseline systemic bone mineral density and annual percentage tibial cartilage volume loss

	Univariable analysis Regression coefficient (95% CI)	*P*	Multivariable analysis^a^ Regression coefficient (95% CI)	*P*
Total body BMD				
Medial	0.02 (−0.26, 0.30)	0.90	0.16 (−0.21, 0.52)	0.40
Lateral	0.22 (0.01, 0.43)	0.04	0.44 (0.17, 0.71)	0.002
Spine BMD				
Medial	0.003 (−0.16, 0.17)	0.98	0.03 (−0.15, 0.20)	0.75
Lateral	0.14 (0.01, 0.26)	0.03	0.17 (0.04, 0.30)	0.01
Total hip BMD				
Medial	0.01 (−0.17, 0.18)	0.95	0.07 (−0.15, 0.29)	0.53
Lateral	0.16 (0.03, 0.30)	0.02	0.29 (0.13, 0.45)	0.001

Bone mineral density (BMD) was assessed in g/10 cm^2^ so that one unit change in BMD approximated one standard deviation change in BMD. ^a^Adjusted for gender, baseline age, body mass index, strenuous physical activity, and respective tibial bone area

There was no evidence of gender or obesity status (yes/no) modifying the association between systemic BMD and cartilage outcomes (all *p* values for interaction > 0.10). When analyses were performed in women only, the magnitude and direction of the results were basically unchanged but some results were no longer statistically significant, most likely due to loss of power for the smaller sample size (Table 5). There were 53 (42%) women who were aged ≥51 years at baseline. Additional adjustment for menopausal status (using the age of 51 years as a surrogate for categorisation) did not change the results (Table 5). Similar results were shown for the two strata when analyses were stratified by obesity and non-obesity (Table 6).

**Table 5:** Association of baseline systemic bone mineral density with progression of tibiofemoral cartilage defects and annual percentage tibial cartilage volume loss in women

	Multivariable analysis^a^ Odds ratio (95% CI)	*P*	Multivariable analysis^b^ Odds ratio (95% CI)	*P*
Progression of tibiofemoral cartilage defects				
Total body BMD				
Medial	1.56 (0.86, 2.84)	0.14	1.80 (0.96, 3.38)	0.07
Lateral	1.36 (0.76, 2.42)	0.30	1.33 (0.73, 2.40)	0.35
Spine BMD				
Medial	1.32 (0.99, 1.78)	0.06	1.38 (1.01, 1.88)	0.04
Lateral	1.30 (0.97, 1. 74)	0.07	1.30 (0.96, 1. 74)	0.09
Total hip BMD				
Medial	1.53 (1.03, 2.28)	0.03	1.74 (1.13, 2.66)	0.01
Lateral	1.42 (0.97, 2.07)	0.07	1.41 (0.95, 2.09)	0.09
	Multivariable analysis^c^ Regression coefficient (95% CI)	*P*	Multivariable analysis^d^ Regression coefficient (95% CI)	*P*
Annual tibial cartilage volume loss (%)				
Total body BMD				
Medial	0.25 (−0.15, 0.65)	0.22	0.29 (−0.12, 0.71)	0.16
Lateral	0.56 (0.20, 0.91)	0.002	0.55 (0.19, 0.92)	0.003
Spine BMD				
Medial	0.06 (−0.14, 0.26)	0.55	0.07 (−0.13, 0.27)	0.50
Lateral	0.23 (0.05, 0.40)	0.01	0.22 (0.04, 0.40)	0.01
Total hip BMD				
Medial	0.20 (−0.05, 0.45)	0.11	0.24 (−0.02, 0.49)	0.07
Lateral	0.41 (0.19, 0.62)	<0.001	0.41 (0.18, 0.63)	<0.001

Bone mineral density (BMD) was assessed in g/10 cm^2^ so that one unit change in BMD approximated one standard deviation change in BMD. ^a^Adjusted for baseline age, body mass index, strenuous physical activity, respective baseline tibial cartilage volume and bone area, and time between scans. ^b^Adjusted for baseline age, body mass index, strenuous physical activity, respective baseline tibial cartilage volume and bone area, time between scans, and menopausal status. ^c^Adjusted for baseline age, body mass index, strenuous physical activity, and respective tibial bone area. ^d^Adjusted for baseline age, body mass index, strenuous physical activity, respective tibial bone area, and menopausal status

**Table 6:** Association of baseline systemic bone mineral density with progression of tibiofemoral cartilage defects and annual percentage tibial cartilage volume loss, stratified by obesity status

	Multivariable analysis^a^ Odds ratio (95% CI) Non-obese	*P*	Multivariable analysis^a^ Odds ratio (95% CI) Obese	*P*
Progression of tibiofemoral cartilage defects				
Total body BMD				
Medial	2.20 (0.65, 7.42)	0.20	2.26 (0.95, 5.37)	0.07
Lateral	1.25 (0.54, 2.89)	0.61	1.43 (0.66, 3.08)	0.37
Spine BMD				
Medial	1.72 (0.92, 3.21)	0.09	2.02 (1.24, 3.29)	0.01
Lateral	1.30 (0.83, 2.02)	0.25	1.13 (0.77, 1.65)	0.52
Total hip BMD				
Medial	2.53 (1.01, 6.32)	0.048	1.71 (1.01, 2.89)	0.047
Lateral	1.52 (0.86, 2.69)	0.15	1.46 (0.90, 2.36)	0.12
	Multivariable analysis^b^ Regression coefficient (95% CI) Non-obese	*P*	Multivariable analysis^b^ Regression coefficient (95% CI) Obese	*P*
Annual tibial cartilage volume loss (%)				
Total body BMD				
Medial	0.05 (−0.40, 0.49)	0.83	0.30 (−0.29, 0.89)	0.31
Lateral	0.53 (0.14, 0.92)	0.01	0.68 (0.19, 1.18)	0.01
Spine BMD				
Medial	0.05 (−0.18, 0.29)	0.66	0.11 (−0.16, 0.38)	0.42
Lateral	0.19 (−0.02, 0.41)	0.08	0.30 (0.06, 0.54)	0.02
Total hip BMD				
Medial	0.02 (−0.26, 0.30)	0.88	0.12 (−0.21, 0.44)	0.47
Lateral	0.27 (0.02, 0.53)	0.03	0.48 (0.21, 0.75)	0.001

Bone mineral density (BMD) was assessed in g/10 cm^2^ so that one unit change in BMD approximated one standard deviation change in BMD. ^a^Adjusted for gender, baseline age, body mass index, strenuous physical activity, respective baseline tibial cartilage volume and bone area, and time between scans. ^b^Adjusted for ender, baseline age, body mass index, strenuous physical activity, and respective tibial bone area

## Discussion

This is the first study to demonstrate that higher systemic BMD is associated with deleterious early structural changes in knee cartilage in middle-aged people without clinical knee disease. Higher systemic BMD was associated with increased progression of cartilage defects in the medial and lateral tibiofemoral compartments and with accelerated loss of lateral tibial cartilage volume.

Our findings of an adverse effect of higher systemic BMD on longitudinal changes to knee cartilage extend the results from two previous cross-sectional studies of predominantly asymptomatic middle-aged adults [16, 17]. These cross-sectional studies showed that higher systemic BMD is associated with greater tibial cartilage volume and a higher prevalence of tibiofemoral cartilage defects [16, 17]. The positive cross-sectional associations with cartilage volume need cautious interpretation because greater cartilage volume may represent cartilage swelling in the early stages of degeneration [18]. Assessing cartilage volume in the context of cartilage defects can help with interpretation of the findings, as cartilage defects are early structural abnormalities that predict cartilage volume loss and radiographic evidence of knee OA [21, 25]. Thus the findings from the two cross-sectional studies [16, 17] suggest an overall detrimental effect of higher systemic BMD in early damage to knee cartilage.

There have been conflicting data from cohort studies on the association between systemic BMD and longitudinal changes to knee cartilage, which might be due to the differences among studies in terms of the characteristics of study participants, outcome measures, or the health status of the knee joint (summarized in Table 1). In people with radiographic knee OA, two previous MRI studies showed that higher systemic BMD was associated with an increase in knee cartilage thickness [1] and that longitudinal BMD loss was associated with increased loss of knee cartilage thickness and volume [26], suggesting a protective effect of maintaining higher systemic BMD against future cartilage loss. In contrast, a radiographic study found no significant association between systemic BMD and progression of joint space narrowing in those with existing radiographic evidence of OA [2]. In people without radiographic evidence of knee OA, a radiographic study demonstrated that high systemic BMD was associated with an increased risk of cartilage loss measured by an increase in the grade of joint space narrowing [2], while an MRI study found no significant association between systemic BMD and change in knee cartilage thickness or volume [1].

Compared with the two previous studies in non-OA populations [1, 2], our study examined a younger population with higher systemic BMD at baseline. We found that higher systemic BMD was associated with both increased cartilage defect progression and accelerated cartilage volume loss, independent of age, BMI, and subchondral bone area. We also found similar results in subgroup analyses of obese and non-obese participants. While our findings were consistent with the results of a previous radiographic study in which higher BMD was a predictor of incident radiological OA [2], our study extended these findings showing that higher systemic BMD predicted early morphological changes to the knee cartilage assessed by MRI, progression of cartilage defects and cartilage volume loss, which are complementary sensitive measures of early cartilage damage. Although the previous MRI study did not find an association between systemic BMD and cartilage loss [1], the moderate sample size of the non-OA population (*n* = 69) may have limited the power of the study to detect a significant relationship, and also there was no adjustment for lower limb bone size, which is an important confounder in assessing the association between areal BMD and knee cartilage changes.

While in our study higher systemic BMD was associated with cartilage defect progression in both the medial and lateral tibiofemoral compartment, the association between systemic BMD and cartilage volume loss was observed in the lateral but not the medial tibia. There is evidence that cartilage defects are an early morphological abnormality of cartilage predating cartilage volume loss [25]. It is likely that progression of cartilage defects is the earlier and more sensitive change in response to higher BMD, compared with cartilage volume loss. Why higher systemic BMD predisposes to loss of volume in the lateral rather than the medial tibial cartilage is not known. Since most of the load during movement is preferentially experienced in the medial compartment [27], it may be that local, rather than systemic BMD is more important in influencing the medial knee cartilage. As the articular cartilage may swell in very early disease [18], this may in part mask an association between higher systemic BMD and volume loss in the medial tibial cartilage, particularly as the medial compartment experiences higher dynamic loads [27]. Some studies have shown that the lateral compartment is more sensitive to change. In people with knee OA, a disease-modifying effect of 2 g/day of strontium ranelate relative to placebo was seen for reducing volume loss in the lateral, but not the medial tibial cartilage at 12, 24, and 36 months [28], and licofelone significantly reduced cartilage volume loss in the lateral rather than the medial compartment at 2 years compared with naproxen [29], suggesting the effects on cartilage volume loss as being in the lateral compartment, as seen in our study.

This study has a number of limitations. It comprised predominantly female participants so studies with a larger proportion of males are required to improve the generalizability of our findings. The direction and magnitude of the results of the study were unchanged when analyses were performed in women only, but some results were no longer statistically significant, most likely due to reduced sample size and thus reduced power. As this study examined middle-aged adults (mean age 46.7 ± 9.3 years), the results are likely to remain generalizable to premenopausal and perimenopausal women, in whom BMD remains preserved relative to the established postmenopausal period. Although we do not have data on anti-resorptive medications that could influence BMD, such as bisphosphonates, this is unlikely to have been a factor, as the cohort were middle-aged, participants with other significant medical conditions were excluded, and none of the participants had osteoporosis. The participants were, in part, recruited through weight-loss clinics and organisations, accounting for the higher average BMI (32.4 ± 8.9 kg/m^2^) and the larger percentage (53.6%) of the cohort being obese. Although this may have led to some selection bias, we have adjusted for BMI in the statistical analyses and performed stratified analyses based on obesity status, which produced very similar results. As obesity is arguably the strongest modifiable risk factor for the development of knee OA [30], our recruitment method was designed to target people most at risk for developing knee OA, improving the clinical applicability of this study. As weight loss retards cartilage damage [31], the inclusion of people attending weight loss clinics who subsequently achieved weight loss would only have reduced our ability to demonstrate significant results relating to cartilage loss and progression of cartilage defects. When we adjusted for weight change, the results remained unchanged (data not shown). Moreover, we have examined people with no diagnosed knee OA. This accounts for the very mild symptoms experienced by this cohort (Table 2). Nevertheless, it is possible that some participants may have had radiographic evidence of knee OA at recruitment. Our analyses of cartilage volume loss and progression of cartilage defects were standardized or adjusted for baseline cartilage volume, which correlates with radiographic joint space width, to control for the baseline joint status. Another limitation is that in our study we did not collect data on spine, hip, or hand OA, which are associated with high systemic BMD.

The strengths of our study include the pre-clinical population with a modest sample size and a wide range of BMI, the sensitive measurements of early cartilage damage (cartilage volume loss and cartilage defect progression) for which we found consistent results, and the adjustment of knee bone size in the statistical analyses.

The mechanism accounting for the associations between high systemic BMD and early damage to knee cartilage is unclear. It may be that higher systemic BMD and structural joint damage share common underlying mechanisms, such as genetic predisposition. Osteoblast and chondrocyte development and function both share the Wnt/B-catenin pathway [32] and abnormalities in this pathway have been identified in the phenotype of high BMD [33, 34] and knee OA [35]. Moreover, higher systemic BMD may not simply be reflective of higher-quality bone. In early knee OA there is increased trabecular thickness and density but relatively decreased connectivity, resulting in reduced mechanical properties of subchondral bone [36], which makes the overlying cartilage more susceptible to damage. It may be that in early knee OA, dysregulated bony proliferation may lead to increased systemic BMD, which may be reflected by localised subchondral bone sclerosis at sites prone to OA, such as the knee. As the knee joints have a broad spectrum of features of subchondral calcified tissue from healthy joint and pre-clinical OA through to established OA [36, 37], the relationship between BMD and articular cartilage may differ throughout the disease course. While previous studies demonstrated a beneficial effect of higher systemic BMD on reducing cartilage loss in older people with knee OA [1, 26] and clinical trials have shown a promising effect of anti-resorptive drugs in reducing the progression of knee OA [28, 38], our findings suggest an adverse effect of higher systemic BMD in early damage to cartilage in middle-aged people with relatively normal BMD and without clinical knee disease. Further work will be required to clarify the effect of systemic BMD at different stages of the pathway from health through to disease.

## Conclusions

This is the first prospective cohort study to demonstrate that higher systemic BMD is associated with increased MRI-detected early damage to knee cartilage in middle-aged individuals without clinical knee disease, evidenced by increased progression of cartilage defects and accelerated cartilage volume loss over 2 years. Further work exploring the effect of systemic versus local BMD, and the influence of BMD on knee structural changes at different stages of the pathway from health through to disease in knee OA, will be important in order to optimize the prevention and treatment of knee OA, particularly as new therapies targeting bone are developed for the management of OA.

## Abbreviations

BMD: Bone mineral density
BMI: Body mass index
CV: Coefficient of variation
DXA: Dual-energy X-ray absorptiometry
MRI: Magnetic resonance imaging
OA: Osteoarthritis
OR: Odds ratio
WOMAC: Western Ontario and McMaster University Osteoarthritis Index
